# Sequencing and *De Novo* Assembly of the Western Tarnished Plant Bug (*Lygus hesperus*) Transcriptome

**DOI:** 10.1371/journal.pone.0055105

**Published:** 2013-01-24

**Authors:** J. Joe Hull, Scott M. Geib, Jeffrey A. Fabrick, Colin S. Brent

**Affiliations:** 1 Pest Management and Biocontrol Research Unit, Agricultural Research Service, United States Department of Agriculture, Maricopa, Arizona, United States of America; 2 Tropical Crop and Commodity Protection Research Unit, Agricultural Research Service, United States Department of Agriculture, Hilo, Hawaii, United States of America; Volcani Center, Israel

## Abstract

**Background:**

Mirid plant bugs (Hemiptera: Miridae) are economically important insect pests of many crops worldwide. The western tarnished plant bug *Lygus hesperus* Knight is a pest of cotton, alfalfa, fruit and vegetable crops, and potentially of several emerging biofuel and natural product feedstocks in the western US. However, little is known about the underlying molecular genetics, biochemistry, or physiology of *L. hesperus*, including their ability to survive extreme environmental conditions.

**Methodology/Principal Findings:**

We used 454 pyrosequencing of a normalized adult cDNA library and *de novo* assembly to obtain an adult *L. hesperus* transcriptome consisting of 1,429,818 transcriptomic reads representing 36,131 transcript isoforms (isotigs) that correspond to 19,742 genes. A search of the transcriptome against deposited *L. hesperus* protein sequences revealed that 86 out of 87 were represented. Comparison with the non-redundant database indicated that 54% of the transcriptome exhibited similarity (*e*-value ≤1^−5^) with known proteins. In addition, Gene Ontology (GO) terms, Kyoto Encyclopedia of Genes and Genomes (KEGG) annotations, and potential Pfam domains were assigned to each transcript isoform. To gain insight into the molecular basis of the *L. hesperus* thermal stress response we used transcriptomic sequences to identify 52 potential heat shock protein (Hsp) homologs. A subset of these transcripts was sequence verified and their expression response to thermal stress monitored by semi-quantitative PCR. Potential homologs of Hsp70, Hsp40, and 2 small Hsps were found to be upregulated in the heat-challenged adults, suggesting a role in thermotolerance.

**Conclusions/Significance:**

The *L. hesperus* transcriptome advances the underlying molecular understanding of this arthropod pest by significantly increasing the number of known genes, and provides the basis for further exploration and understanding of the fundamental mechanisms of abiotic stress responses.

## Introduction

As a polyphagous piercing-sucking pest with a documented host plant range in excess of 150 species, the western tarnished plant bug, *Lygus hesperus* Knight, causes economic losses in numerous cropping systems in western North America. *L. hesperus* is a multivoltine species with a geographic range that extends from southern Mexico to the southwestern provinces of Canada. After release from a reproductive diapause induced by a short photophase [1]–[3] adults colonize early flowering host plants [4] and subsequently disperse in multi-generational waves throughout the growing season to traditional crops such as cotton, strawberries, and alfalfa [5], [6] as well as emerging biofuel feedstocks [7]–[11]. Control strategies have traditionally relied on broad-spectrum insecticides; however, ecological ramifications and the presence of insecticide resistance in *L. lineolaris* field populations [12]–[14] have limited the arsenal available for effective *Lygus* management.

The ability of wild *L. hesperus* populations to persist in the arid conditions (ambient air temperatures that exceed 42°C and relative humidity below 10%) of the southwestern US is determined by the thermal sensitivities of key traits such as development, life span, and fecundity. The induction of heat shock proteins (Hsps), which provide cellular protection against the deleterious effects of thermal stress, have been reported to impact a number of these traits (reviewed in [15]–[17]). Hence, we are interested in identifying the molecular pathways involved in *L. hesperus* thermotolerance and elucidating the phenotypic plasticity that allows them to occupy a wide variety of environments.

Discovery of the underlying biochemical and physiological mechanisms used by *L. hesperus* to adapt to adverse environments remains challenging. Indeed, the current paucity of molecular data and understanding of *L. hesperus* thermal sensitivity is insufficient for producing accurate predictive models of dispersal and population growth. To begin to address this issue, we *de novo* assembled and annotated a comprehensive transcriptome for adult *L. hesperus* using second-generation pyrosequencing data. Similar efforts utilizing various next generation platforms have been successfully used to *de novo* assemble a number of non-model insect transcriptomes including poplar leaf beetle (*Chrysomela tremulae*) [18], tobacco hornworm (*Manduca sexta*) [19], soybean aphid (*Aphis glycines*) [20], two whiteflies (*Bemisia tabaci* and *Trileurodes vaporariorum*) [21], [22], oriental fruit fly (*Bactrocera dorsalis*) [23], brown planthopper (*Nilaparvata lugens*) [24], walking stick (*Timema cristinae*) [25], blow fly (*Lucilia sericata*) [26], housefly (*Musca domestica*) [27], and mountain pine beetle (*Dendroctonus ponderosae*) [28]. We assessed the functional quality of the transcriptome by identifying genes potentially involved in mediating *L. hesperus* thermotolerance, and examined the expression profile of a subset of those genes in adult females following exposure to thermal stress conditions.

## Results and Discussion

### Transcriptomic analysis

**Figure 1:**
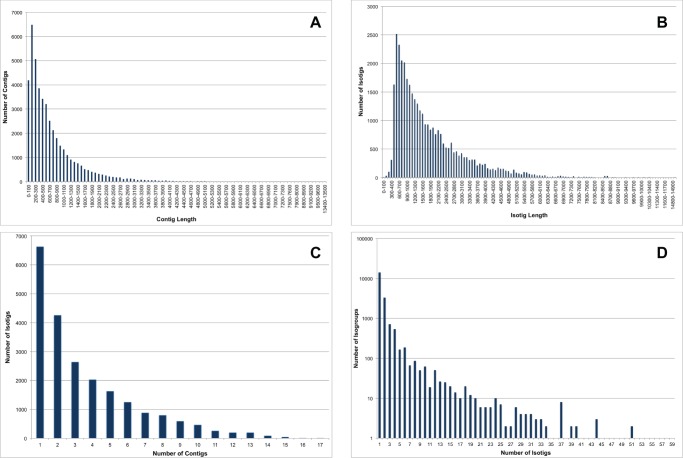
Summary of mixed sex adult *L. hesperus* transcriptomic sequences. (A) Length distribution of contig sequences. (B) Length distribution of isotig sequences. (C) Number of contigs used in the assembly of individual isotigs. (D) Number of isotigs used in the assembly of individual isogroups.

To develop a more comprehensive understanding of the molecular mechanisms governing *L. hesperus* biology, we performed Roche 454 pyrosequencing of a normalized cDNA library prepared from 20 mixed sex adults aged 0–5 days post-eclosion. Sequencing generated 1,429,818 transcriptomic reads consisting of 561,933,830 bp. After removal of adaptor sequences, data were aligned and *de novo* assembled using version 2.6 of the newbler assembler (454 Life Sciences/Roche, Branford, CT) into 44,505 contigs consisting of 32,252,977 bp. Contigs ranged in size from 2–13,480 bp with an average length of 725 bp (Figure 1A). The contigs were then assembled into 36,131 potential transcript splice variants (referred to as isotigs) that had an average size of 1,793 bp (Figure 1B). While 14,059 isotigs were derived from single contigs, the average number of contigs per isotig was 3.2 with the highest consisting of 18 contigs (Figure 1C). The isotigs were further assembled by the newbler software into 19,746 isogroups, which potentially correspond to the total number of genes expressed in the adult *L. hesperus* transcriptome. A total of 14,187 isogroups contained only a single isotig, although on average there were 1.8 isotigs per isogroup (Figure 1D).

To assess the potential completeness and quality of the transcriptome, we used publically available *L. hesperus* protein data to perform a tBLASTn analysis, which searches a translated nucleotide database with a protein query sequence. The NCBI database (Sept. 2012) listed 88 protein sequences from *L. hesperus* including one erroneously listed *Bacillus thuringiensis* parasporal crystal protein (ADK94873). After removal of this protein from the data set, we found that only one of the deposited sequences (AEK80439; a putative sex peptide receptor) was not represented in the transcriptome. The remaining sequences had *e*-values of 0.0 to 3.9*e* ^−38^ and sequence identities of 80–100% (Table S1). The variation observed between the deposited sequences and those in the transcriptome likely reflect the genetic composition of the *L. hesperus* colony utilized to generate the transcriptome as well as genetic variation in the insects from which the deposited sequences were derived. Because the deposited data included a number of duplications, we found that the deposited proteins ultimately corresponded to only eight different genes.

**Figure 2:**
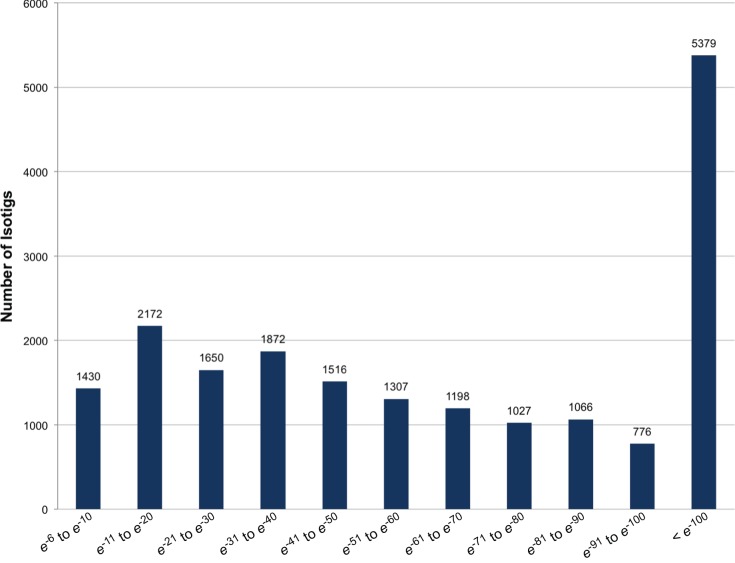
Distribution of BLASTx homology search *e*-values. BLAST analysis against the non-redundant database was performed with assembled *L. hesperus* isotig sequences and an *e*-value cutoff of 1*e* ^−5^.

**Figure 3:**
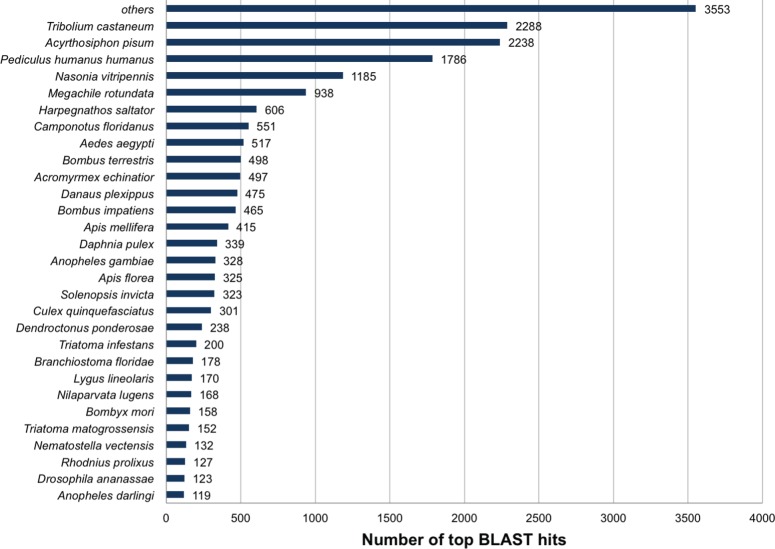
Species distribution of the top *L. hesperus* isotig sequence BLASTx hits. BLAST analysis against the non-redundant database was performed with an *e*-value cutoff of 1*e* ^−5^.

BLASTx analysis (cutoff *e*-value <1*e* ^−5^) of the isotigs indicated that 19,393 of the sequences (54%) were homologous to proteins in the non-redundant database (Table S2). The remaining sequences lacked an *e*-value below the cutoff, suggesting that they may comprise novel genes specifically expressed in *L. hesperus*. Alternatively, these sequences may correspond to untranslated regions or errors in isotig/contig assembly. The percentage of *L. hesperus* isotig sequences with homology to known proteins is consistent with that reported for brown planthopper (*N. lugens*) (56%) [29], oriental fruit fly (*B. dorsalis*) (55%) [23], pine shoot beetle (*Tomicus yunnanensis*) (60%) [30], and soybean aphid (*A. glycines*) (42%) [20], and is considerably higher than that reported for other non-model insect pests whose transcriptomes were likewise profiled using Roche 454 methodologies [31]–[33]. Further analysis of the BLAST data indicated that nearly a third of the top BLASTx hits had *e*-values <*e* ^−100^ (Figure 2) and that the highest percentage (12%; 2,288 isotigs) of hits exhibited significant similarity with sequences identified in the red flour beetle (*Tribolium castaneum*) (Figure 3). While *Tribolium* and *Lygus* are from different orders (Coleoptera and Hemiptera, respectively), and therefore phylogenetically distinct, the finding that the most abundant top BLASTx hits were from the red flour beetle is not entirely unexpected given the enormity of available *Tribolium* sequences and completeness of annotation in this species. Species with the next most abundant BLASTx similarities included the pea aphid (*Acyrthosiphon pisum*) and the human body louse (*Pediculus humanus humanus*), two heterometabolous arthropod species that along with *L. hesperus* comprise a portion of the hemipteroid assemblage. The relatively high number of sequences exhibiting similarity with proteins from non-insect species (a water flea, *Daphnia pulex*; a lancelet, *Branchiostoma floridae*, and a sea aneomone, *Nematostella vectensis*), representing three distinct phylums, may provide evolutionary clues regarding the conservation of potentially ancestral genes.

### Comparative analysis

**Figure 4:**
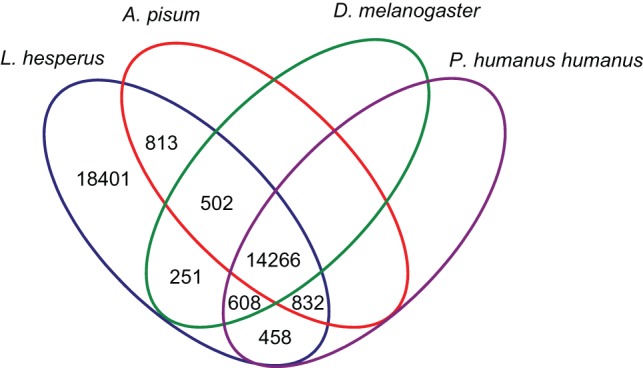
Comparative summary of *L. hesperus* isotig sequences with protein sequences from three insect species. Amino acid sequence comparisons were performed using BLASTx with predicted protein sequences from the holometabolous insect, *Drosophila melanogaster* (Diptera), and two representatives of the hemipteran assemblage, *Acyrthosiphon pisum* (pea aphid) and *Pediculus humanus humanus* (human body louse).

Comparison of the translated *L. hesperus* transcriptome with draft protein sequences of *A. pisum* (Hemiptera), *P. humanus humanus* (Phthiraptera), and *Drosophila melanogaster* (Diptera) revealed comparable sequence similarity across species. Approximately 45% (i.e., 16413 BLAST hits) of the 36,131 transcriptomic sequences from *L. hesperus* exhibited significant similarity with proteins in *A. pisum*, another 45% (16,164 BLAST hits) with proteins in *P. humanus humanus*, and 43% (15,627 BLAST hits) with proteins in *D. melanogaster* (Figure 4). While 14,266 sequences were shared amongst the four insects (Table S3), *L. hesperus* shared a greater number of unique sequences with *A. pisum* (813) than either *P. humanus humanus* (458) or *D. melanogaster* (251). Nearly 65% of the *L. hesperus* sequences had no BLASTx similarity with the three species, suggesting that they may encode novel proteins, represent untranslated regions, or correspond to incorrectly assembled contigs.

### Gene ontology

**Figure 5:**
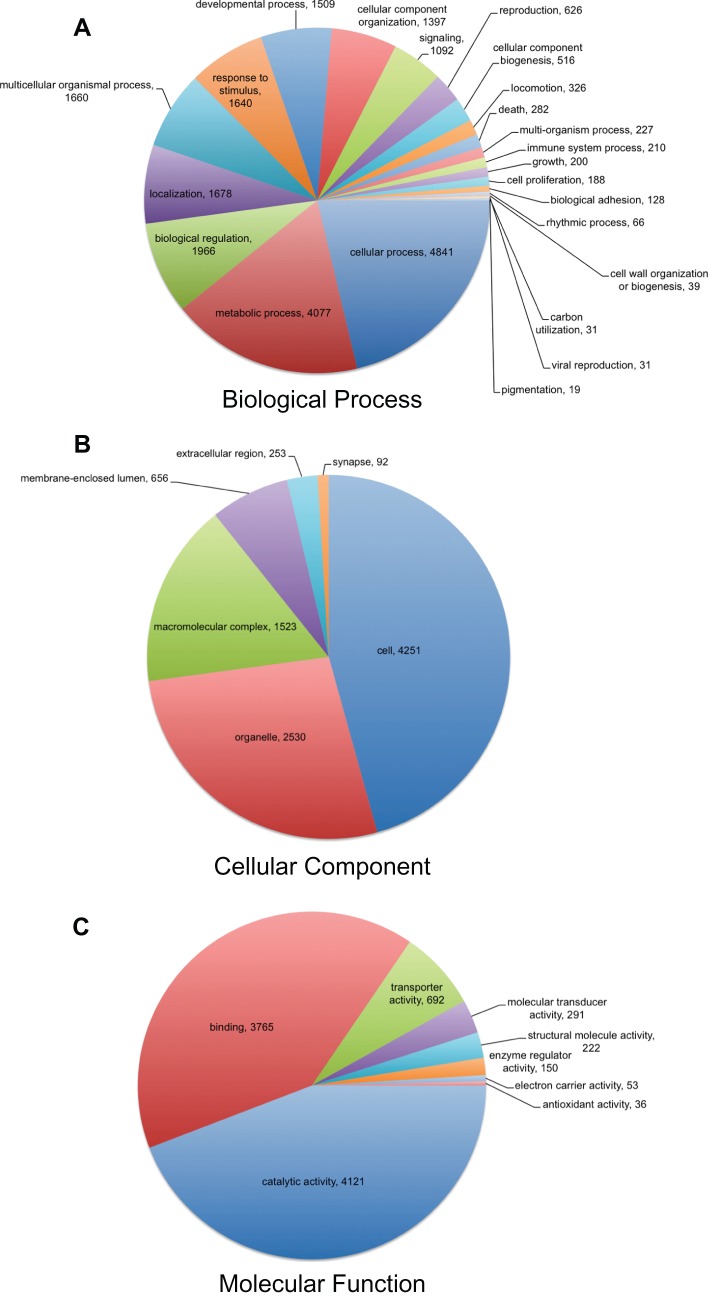
Classification of *L. hesperus* isotig sequences based on predicted Gene Ontology (**GO**) **terms.** (A) Biological Process, (B) Cellular Components, and (C) Molecular Function. GO terms were determined using Blast2GO [34], [35] with an *e*-value cutoff of 1*e* ^−5^, a 10% initial filter, and sorted based on level 2 classifications.

To facilitate organization of the *L. hesperus* transcripts into putative functional groups, Gene Ontology (GO) terms were assigned using Blast2GO [34], [35] (Table S4). A total of 7,898 isotigs were assigned GO terms, including 5,961 sequences at the biological process level (Figure 5A), 4,413 sequences at the cellular component level (Figure 5B), and 6,445 sequences at the molecular function level (Figure 5C). The distribution of GO terms within the ontology categories is consistent with other insect transcriptomes [30]–[32], [36]. Within the Biological Process GO category, isotigs assigned to cellular (4,841) and metabolic processes (4,077) were most abundant (Figure 5A). Cell (4,251) and organelle (2,530) terms were most abundant within the Cellular Component category (Figure 5B). For Molecular Function, isotig sequences were predominantly assigned to catalytic activity (4,121) and binding (3,765) functions (Figure 5C). Intriguingly, the number of *L. hesperus* isotigs assigned with putative antioxidant activity (n = 36) within the Molecular Function GO category, was higher as a percentage (0.56%) than that found in other insects (0.02–0.4%) [22], [30]–[32], [36]. As oxidative damage has been linked to thermal stress [37], the increased expression of antioxidant-related transcripts in *L. hesperus* may reflect a genetic mechanism that provides some measure of thermotolerance to the extreme climatic conditions of the arid southwestern US.

### Metabolic pathways

**Figure 6:**
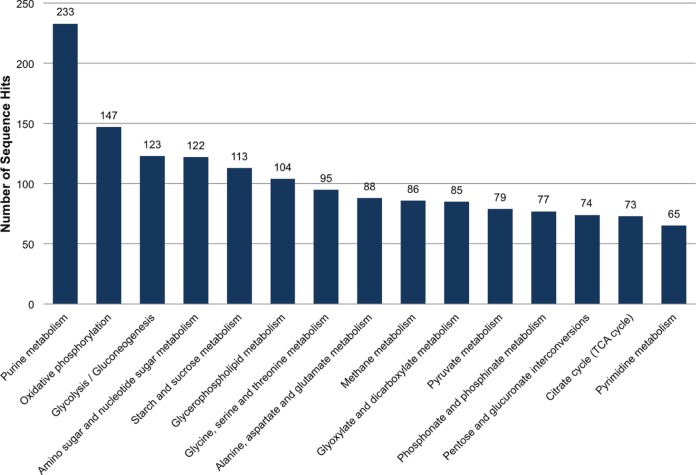
Distribution of *L. hesperus* isotig sequences among KEGG (**Kyoto Encyclopedia of Genes and Genomes**) **pathways.** The top 15 most highly represented pathways are shown. Analysis was performed using Blast2GO and the KEGG database [38], [39].

We used the Kyoto Encyclopedia of Genes and Genomes (KEGG) database [38], [39] to identify potential pathways represented in the transcriptome. Based on comparative analyses, we assigned 3,271 sequences to 114 KEGG pathways with metabolic processes (purine metabolism, oxidative phosphorylation, glycolysis, etc) most highly represented (Figure 6; Table S5). This investment in metabolic transcripts may reflect maintenance of a higher metabolic rate in response to elevated temperatures [40]. Only six sequences were associated with cytochrome P450-induced drug/xenobiotic metabolism, which often functions in insecticide resistance. This total is somewhat surprising given the preponderance of cytochrome P450-associated domains in the transcriptome (see below), and illustrates the limitations of drawing conclusions about gene functionality based on data that is largely descriptive and built on models derived from unrelated organisms.

### Protein domains

**Figure 7:**
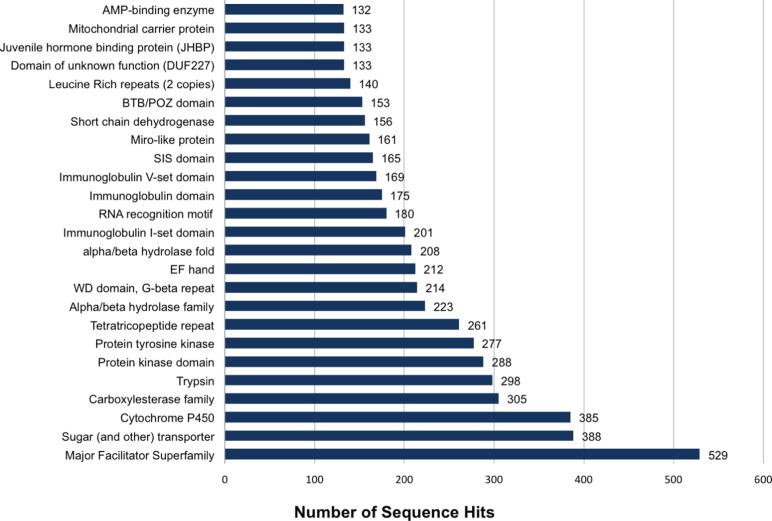
Distribution of the top 25 Pfam domains identified in translated *L. hesperus* sequences. Analysis was performed using HMMER3 [69] with Pfam database A.

A Pfam domain database search identified 32,036 instances of 3,705 protein domains in 16,671 isotig sequences (Table S6). The most abundant domains identified (Figure 7) were those found in sugar transporters and members of the major facilitator superfamily: a ubiquitous group of integral membrane proteins involved in the transport of diverse substrates (ions, neurotransmitters, amino acids, peptides, and drugs). Multiple transport mechanisms have been linked with the major facilitator superfamily including transport of a single substrate along a concentration gradient (uniport), transport of multiple substrates in the same direction using the concentration gradient of one as the driving force (symport), and transport of multiple substrates in opposite directions (antiport) [41].

Protein domains often associated with detoxification enzymes (cytochrome P450s and carboxylesterase) were also among the most highly represented domains in the transcriptome. Cytochrome P450s are a diverse superfamily of enzymes crucial to the metabolization of a wide array of plant-derived allelochemicals and insecticides [42]. Carboxylesterases are hydrolases that catalyze the cleavage of ester bonds in organic carboxylic acids and are linked with insecticide resistance [43]. A subset of cytochrome P450s and carboxylesterases were shown to be upregulated in response to acephate exposure in *L. lineolaris* [44], which may provide a potential molecular basis for resistance in this species [12]–[14].

Other highly represented protein domains included those associated with hydrolytic enzymes (trypsins and alpha/beta hydrolases), cellular signaling (protein and tyrosine kinases), and immunoglobin-like domains. Comparison of Pfam searches between *L. hesperus*, *C. lectularius*, and *A. glycines* [20], [32], indicated an overall similarity of identified domains, with 30 of the respective top 75 domains shared (Figure S1).

### Identification of heat shock proteins

Perturbations of only a few degrees outside of an organism's usual temperature range can disrupt cellular homeostasis and profoundly impact development, fecundity, and longevity [15]–[17]. The deleterious effects of thermal stress are principally caused by impaired protein folding, which can lead to protein inactivation and/or indiscriminate protein-protein interactions that can negatively impact the organization of the cytoskeleton, intracellular transport, RNA splicing, oxidative phosphorylation, and membrane permeability [45]. Thermal stress can trigger a number of cellular responses that function to minimize and abrogate the deleterious effects of the stress, the most predominant of which is the elevated expression of heat shock proteins (Hsps). Hsps are a group of highly conserved, yet highly diversified (10–90 kDa) proteins that primarily function as molecular chaperones, stabilizing protein folding and preventing indiscriminate protein interactions by sequestering unfolded proteins [15].

**Figure 8:**
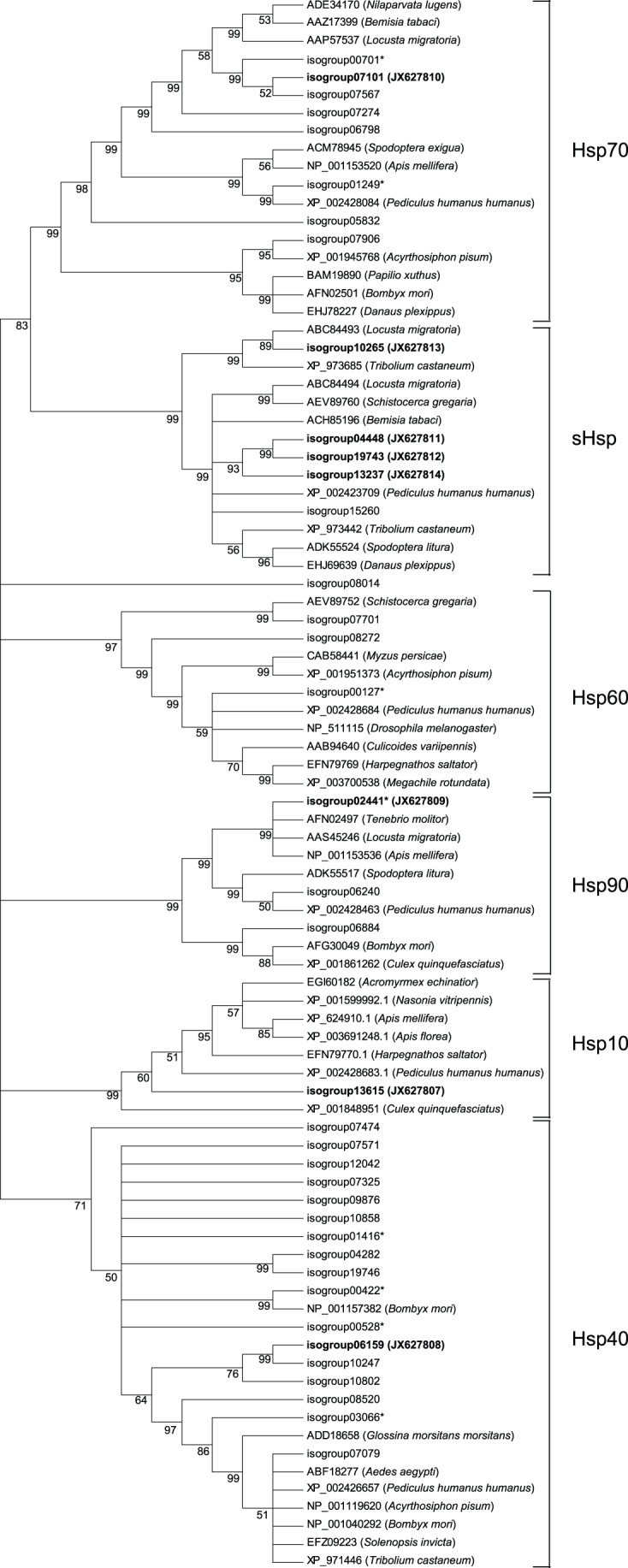
Phylogenetic relationships between predicted *L. hesperus* heat shock protein (**Hsps**) **sequences and Hsps from various insect species.** Sequences were aligned using MAFFT with default settings. The phylogenetic tree was constructed using the maximum parsimony method implemented in MEGA 5 [72] with default settings. Percentage bootstrap support values above 50%, based on 1000 replicates, are shown. Analyses were performed using only sequences predicted to encode complete ORFs. Non-*L. hesperus* sequences are indicted by accession numbers with the respective genus and species shown in parentheses. *L. hesperus* sequences are indicated by the corresponding isogroup identifier. Sequence-confirmed *L. hesperus* Hsps are shown in bold with the respective accession number in parentheses. Sequences clustered into the six major Hsp protein families (brackets). Asterisk (*) indicates isogroups generated from multiple identical isotig sequences: isogroup00528 = 9 isotigs, isogroup00422 = 9 isotigs, isogroup01416 = 2 isotigs, isogroup03066 = 2 isotigs, isogroup00127 = 12 isotigs, isogroup00701 = 5 isotigs, isogroup01249 = 4 isotigs, and isogroup02441 = 2 isotigs.

To begin to elucidate the molecular basis of thermal stress tolerance in *L. hesperus*, we sought to identify sequences in the transcriptome that encode Hsps. Based on sequence conservation (BLASTx cutoff *e*-value of E<1*e* ^−10^), we identified 89 putative Hsp isotig sequences corresponding to 52 unique genes, referred to as isogroups. Of these isogroups, 38 contained complete open reading frames (ORFs) with putative start and stop codons, and 14 corresponded to partial sequences. While individual isogroups were predominantly generated from a single isotig sequence, eight isogroups (isogroup00127, 00528, 00422, 00701, 01249, 01416, 03066, and 02441)) were found to be derived from multiple isotigs, ranging from two (isogroups 01416, 03066, and 02441) to 12 sequences (isogroup00127). Phylogenetic analysis of the individual isotigs (or, where appropriate, a representative isotig of a multi-isotig cluster) revealed that the *L. hesperus* sequences segregated into clades corresponding to six Hsp families: Hsp10, small Hsps (sHsps), Hsp40, Hsp60, Hsp70, and Hsp90 (Figure 8).

Hsp10 is a 10-kDa chaperone, analogous to the bacterial GroES subunit [46] that functions as a co-chaperone with Hsp60. In bacteria, GroES acts as a lid that covers GroEL (Hsp60) encapsulated proteins, effectively sequestering unfolded proteins and preventing the formation of non-specific protein aggregates. Hsp10 is predominantly a mitochondrial protein, but has been found to localize to a number of cellular compartments. In mammalian systems, Hsp10 has been linked with diverse physiological functions [47]. The role of Hsp10 in insects, however, has not been as clearly defined. Analysis of the *L. hesperus* transcriptome indicated the presence of a single gene transcript (isogroup13615) encoding a complete Hsp10 ORF. Comparison with other insect Hsp10 proteins indicated moderate (66–76%) sequence conservation (Figure S2).

The sHsp family is a heterogeneous group of proteins of intermediate molecular weight (12–43 kDa) that are typified by a core alpha-crystallin domain of 80–90 residues flanked by amino- and carboxyl-terminal regions of variable size and sequence conservation [48]. They function as ATP-independent chaperones by forming large multimeric complexes of homo or hetero-oligomers that interact with and prevent the indiscriminate aggregation of denatured proteins [45]. Recently, various sHsps have been implicated as potential climatic adaptation genes [49], [50]. The most extensive sHsp gene repertoire identified to date is in the silkmoth (*Bombyx mori*), which has 16 sHsp genes. In contrast, *D. melanogaster* has 11 genes, while *Apis mellifera* and *T. castaneum* have 10 genes each, and *Anopheles gambiae* has 7 [51]. Despite these high numbers, evolutionary conservation of sHsps appears to have been restricted to a single orthologous gene with the other sHsp genes arising from species-specific lineages [51]. BLAST analysis of the *L. hesperus* transcriptome identified nine isotig sequences corresponding to nine separate gene products (i.e., isogroups) homologous with sHsps. Five of these sequences (isogroups 15260, 04448, 19743, 10265, and 13237) were found to encode complete ORFs (Table 1). The translated *L. hesperus* sHsp sequences represent highly divergent proteins, with sequence identities ranging from 18–91% (Figure S3). Comparison of *L. hesperus* sHsps with those from other insects also show relatively poor sequence conservation (Figure S3). However, one *L. hesperus* sHsp (isogroup10265) is highly conserved with other insect sHsps and clustered in a separate clade with homologs of the presumptive ancestral sHsp gene (Figure 8).

**Table 1:** Putative *L. hesperus* heat shock protein (Hsp) transcripts.

Hsp Family	Isogroup^1^	Isotig	Number of Isotigs
Hsp10	isogroup13615	isotig29860	1
sHsp	*isogroup03809* 2	*isotig18306*	1
	isogroup04448	isotig19584	1
	isogroup19743	isotig19585	1
	*isogroup05449*	*isotig21586*	1
	*isogroup19744*	*isotig21587*	1
	isogroup10265	isotig26510	1
	isogroup13237	isotig29482	1
	isogroup15260	isotig31505	1
	*isogroup19072*	*isotig35317*	1
Hsp40	isogroup00422	isotig07420, isotig07421, isotig07422, isotig07423, isotig07424, isotig07425, isotig07426, isotig07427, isotig07428	9
	isogroup00528	isotig08285, isotig08286, isotig08287, isotig08288, isotig08289, isotig08290, isotig08291, isotig08292, isotig08293	9
	isogroup01416	isotig12622, isotig12623	2
	isogroup03066	isotig16820, isotig16821	2
	*isogroup04110*	*isotig18908*	1
	*isogroup19745*	*isotig18909*	1
	isogroup04282	isotig19252	1
	isogroup19746	isotig19253	1
	*isogroup05784*	*isotig22029*	1
	isogroup06159	isotig22404	1
	isogroup07079	isotig23324	1
	isogroup07325	isotig23570	1
	isogroup07474	isotig23719	1
	isogroup07571	isotig23816	1
	isogroup08014	isotig24259	1
	isogroup08520	isotig24765	1
	*isogroup08627*	*isotig24872*	1
	*isogroup08698*	*isotig24943*	1
	isogroup09876	isotig26121	1
	isogroup10247	isotig26492	1
	isogroup10802	isotig27047	1
	isogroup10858	isotig27103	1
	isogroup12042	isotig28287	1
	*isogroup16626*	*isotig32871*	1
Hsp60	isogroup00127	isotig03550, isotig03551, isotig03552, isotig03553, isotig03554, isotig03555, isotig03556, isotig03557, isotig03558, isotig03559, isotig03560, isotig03561	12
	isogroup07701	isotig23946	1
	*isogroup08155*	*isotig24400*	1
	isogroup08272	isotig24517	1
Hsp70	isogroup00701	isotig09456, isotig09457, isotig09458, isotig09459, isotig0946	5
	isogroup01249	isotig11985, isotig11986, isotig11987, isotig11988	4
	isogroup05832	isotig22077	1
	isogroup06798	isotig23043	1
	isogroup07101	isotig23346	1
	isogroup07274	isotig23519	1
	isogroup07567	isotig23812	1
	*isogroup07851*	*isotig24096*	1
	isogroup07906	isotig24151	1
	*isogroup09578*	*isotig25823*	1
	*isogroup14384*	*isotig30629*	1
Hsp90	isogroup02441	isotig15625, isotig15626	2
	isogroup06240	isotig22485	1
	isogroup06884	isotig23129	1
**Total**	**52– isogroups (38 full ORFs; 14 partial sequences)**		**89– isotigs**

1 isogroups (genes) were generated from isotigs (potential splice variants) with the same core sequence.

2 partial sequences shown in *italics*.

Members of the Hsp40 family (also referred to as the DnaJ family) are crucial co-factors/co-chaperones of the Hsp70-mediated ATPase activity, essential for stabilizing interactions between Hsp70 and unfolded proteins [45], [52]. The Hsp40 “J” domain, which is required for stimulating Hsp70 ATP hydrolysis, consists of 70 amino acid residues that are frequently located proximal to the Hsp40 amino-terminus. Hsp40 proteins can be sub-grouped into one of three types based on the presence of additional conserved domains/regions. Type I Hsp40 proteins contain the J domain as well as a Gly/Phe rich region and multiple cysteine repeats, type II proteins lack these cysteine repeats, while only the J domain is present in type III proteins [52], [53]. The number of DnaJ binding domain proteins encoded within respective insect genomes is extensive with 50 in *D. melanogaster* and 34 in *A. gambiae*. We identified 42 isotig sequences corresponding to 24 unique Hsp40 isogroups encoding 18 complete ORFs (Table 1). Two of the isogroups (00422 and 00528) were composed of nine isotig sequences each. Because these isotig clusters had identical coding sequences, they were considered to be single Hsps and were thus assigned to the respective isogroups. Analysis of the domain architecture in the isogroup sequences indicated that the *L. hesperus* Hsp40 repertoire consists of four type I proteins, seven type II proteins, and seven type III proteins. The J domain of all but one Hsp40 (isogroup00528) was found near the amino terminus. Sequence conservation amongst the putative *L. hesperus* Hsp40 proteins was predominantly low with most exhibiting <25% sequence identity, although some sequences exhibited moderate to high conservation (51–92%) (Figure S4). Comparison with sHsps from other insects likewise indicates limited sequence conservation for *L. hesperus* sHsps (Figure S4).

Hsp60 proteins form multimeric complexes called chaperonins that function in conjunction with Hsp10 to modulate protein folding under both normal and stress conditions [45], [54]. They are conserved across all taxa and have been extensively studied in bacteria as part of the GroE operon [46]. Hsp60 proteins prevent indiscriminant aggregation of unstructured proteins by forming a double-ring cylindrical oligomer that sequesters unfolded proteins in an environment free from potential spurious interactions that can result in aggregation. ATP-dependent binding of Hsp10 completes the sequestration process. Unlike bacteria, which possess a single Hsp60 protein (GroEL), eukaryotic organisms generally express two types of Hsp60-based chaperonins. Group I chaperonins are analogous to the bacteria GroEL and are localized to the mitochondrial matrix, whereas group II (e.g., chaperonin containing tailless complex polypeptide 1) are cytosolic [55]. Based on BLAST data, we identified 15 isotig sequences corresponding to four Hsp60 isogroups. Isogroup00127 was generated from 12 isotigs containing identical coding sequence information. The other isogroups were each generated from single isotig sequences. Three of the isogroups encode complete ORFs (Table 1). Domain analysis of the amino-terminal portions of the sequences identified mitochondrial targeting motifs (as defined by the online prediction algorithm MitoProt [56]) in isogroups 00127, 08155, and 08272. All three isogroups were most similar to other insect mitochondrial Hsp60 proteins. Isogroup07701 lacked any definable mitochondrial sequence and was most similar to the group II chaperonins. Overall, sequence identity among the translated *L. hesperus* Hsp60s varied from 19–55%, with the three putative mitochondrial proteins sharing the highest similarity (Figure S5). The putative mitochondrial Hsp60s also aligned with a clade separate from the cytosolic Hsp60 (Figure 8). Comparison with orthologous sequences from other insect species indicates that the respective genes are evolutionarily conserved (Figure S5).

The Hsp70 family is the most structurally and functionally conserved group of chaperon proteins [57]. Hsp70 proteins function in routine *de novo* protein folding under normal conditions. However, under stressed conditions they prevent indiscriminant protein aggregation by tightly binding denatured proteins. This process is facilitated by Hsp40, which delivers unstructured proteins to Hsp70 and mediates the ATP hydrolysis necessary for high affinity binding [45]. In eukaryotes, multiple genes have been identified that encode Hsp70 proteins with varying expression patterns and intracellular localizations (e.g., cytsol, mitochondria, endoplasmic reticulum). We identified 18 isotig sequences corresponding to 11 isogroups with high homology to Hsp70 proteins. Complete ORFs were detected in eight of the isogroup sequences (Table 1). Isogroup00701 was generated from five identical isotigs and isogroup01249 from four identical isotigs. Inspection of the translated sequences suggests that four isogroups (00701, 07101, 07274, and 07567) encode cytosolic (carboxyl-terminal EEVD/E sequence) Hsp70 proteins, isogroup01249 encodes a mitochondrial (79% probability based on MitoProt II) Hsp70, and isogroup06798 encodes an endoplasmic reticulum (carboxyl-terminal KDEL sequence) localized Hsp70. Consistent with these predictions, the *L. hesperus* Hsp70 proteins largely clustered with similarly targeted proteins in the phylogenetic analysis (Figure 8). A variation (MVDE) of the carboxyl-terminal EEVD/E motif was identified in isogroup05832, although whether it localizes to the cytosol remains to be empirically demonstrated. No readily recognizable cellular signals were identified in isogroup07906. The remaining isogroups represent incomplete sequences and were too short for analysis. Sequence identities ranged from 4–83% among the putative *L. hesperus* Hsp70 proteins, and from 7–96% with orthologous sequences from other insect species (Figure S6).

Unlike the other Hsps, Hsp90 has been shown to be an extremely abundant protein under normal physiological conditions [45], [58]. Hsp90 is essential in a number of eukaryotic organisms with multiple genes encoding isoforms that localize to various intracellular organelles. Under normal conditions, Hsp90 contributes to the proper folding of various cytosolic proteins including a number of proteins involved in signal transduction cascades [45], [58]. Under heat stress, interactions between Hsp90 and various co-chaperones are thought to lock Hsp90 into a conformation that allows it to function as a sequestration unit, preventing indiscriminant aggregation of denatured proteins [45]. Even though the transcriptional response of Hsp90 to thermal stress is moderate [45], [58], it is an integral component of the cellular defense mechanism. Mutations that affect Hsp90 functionality or expression have been linked to impaired high temperature growth in both yeast and vertebrate cell cultures [59]–[61]. Based on sequence similarities, we identified 4 isotig sequences corresponding to 3 Hsp90 genes (isogroups 02441, 06240, and 06884), all of which contained complete ORFs (Table 1). Isogroup02441 was derived from two identical isotig sequences. Analysis of the three putative *L. hesperus* Hsp90 proteins for cellular signals suggests that they localize to the cytosol, endoplasmic reticulum, and mitochondria, respectively. Consistent with the unique localization signals, sequence conservation was modest among the three Hsp90s (26–42%), and across orthologous sequences from other insect species (25–88%) (Figure S7).

### Validation of transcriptomic Hsp sequences

To further assess the quality of the transcriptome, we sought to confirm the sequence assembly for eight of the predicted Hsp transcripts. All of the selected transcripts were predicted to contain complete ORFs and included potential homologs of four sHsps (Hsp23.6, isogroup13237; Hsp21.9, isogroup19743; Hsp21.5, isogroup10265; and Hsp21.4, isogroup04448), and one isoform each of Hsp10 (isogroup13615), Hsp40 (isogroup06159), Hsp70 (isogroup07101), and Hsp90 (isogroup02441). Full-length sequences for each were cloned from adult female *L. hesperus* whole body cDNAs and compared with the assembled sequences. In all cases, sequence variation was minimal (>97% nucleotide identity) with most variations the result of synonymous mutations (Table 2). The observed discrepancies are likely attributable to allelic variation associated with the heterogeneity of the *L. hesperus* colony, which was annually outbred with local conspecifics, although rare PCR-induced errors may also have contributed. The consensus sequence data for the selected genes have been deposited with GenBank under the accession numbers JX627807-14.

**Table 2:** Validation of select transcriptome Hsp sequences (isogroup *vs*. consensus cloned sequence).

Isogroup	Cloned Gene	Accession No.	Nucleotide Identity (%)	Number Synonymous Mutations per ORF (nt)	Number Nonsynonymous Mutations per ORF (nt)	Amino acid Identity (%)
isogroup13615	LhHsp10	JX627807	>99%	2/318	1/318	>99%
isogroup10265	LhHsp21.5	JX627813	>99%	1/573	0/573	100%
isogroup04448	LhHsp21.4	JX627811	>99%	2/576	1/576	>99%
isogroup19743	LhHsp21.9	JX627812	>99%	2/624	0/624	100%
isogroup13237	LhHsp23.6	JX627814	>97%	9/588	1/588	>99%
isogroup06159	LhHsp40-1	JX627808	>99%	2/1056	1/1056	>99%
isogroup07101	LhHsp70-1	JX627810	>98%	27/1932	2/1932	>99%
isogroup02441	LhHsp90-1	JX627809	>99%	17/2169	7/2169	>99%

**Figure 9:**
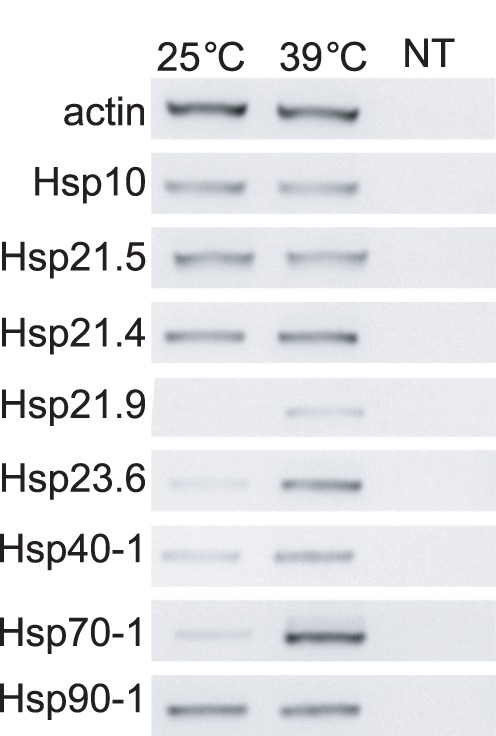
Semi-quantitative expression analyses of eight *L. hesperus* Hsps in response to thermal stress. PCR was performed using cDNA prepared from 6-day old adult *L. hesperus* females exposed for 6 hr to either normal conditions (25°C) or thermal stress conditions (39°C). Actin was used as an amplification control. Products were analyzed on 1.5% agarose gels and stained with SYBR Safe. For clarity, the negative images of the gels are shown. Amplification data are representative of three biological replicates.

To begin to assess the affect of thermal stress on *L. hesperus*, we used semi-quantitative end-point PCR to compare transcript profiles of the above Hsps from adult females under normal (25°C) and stress (39°C for 6 hr) conditions. The lower temperature was used to rear the insects from egg to adult, while the higher temperature was set 2°C below the lethal threshold observed for this laboratory-reared population. The effects of thermal stress on transcription were gene dependent. Hsp70-1 (JX627810) and Hsp23.6 (JX627814) show robust increase in expression, whereas Hsp40-1 (JX627808) and Hsp21.9 (JX627812) exhibit only a moderate increase (Figure 9). In contrast, no changes in transcript levels were observed for Hsp10 (JX627807), Hsp21.5 (JX627813), Hsp21.4 (JX627811), and Hsp90-1 (JX627809). There was likewise no change in expression with the non-heat inducible control gene, actin. These results provide a clear demonstration that exposure to elevated temperature induces a transcriptional Hsp response in *L. hesperus*, and provides further validation regarding the utility of the transcriptomic data. While the affects of thermal stress on *L. hesperus* Hsp transcription varied (i.e., not all Hsp transcripts change in response to heat stress), our results are consistent with other reports indicating that the threshold underlying the induction of Hsp expression is gene dependent [15]. Future high-throughput next-generation sequencing experiments will enhance our understanding of the *L. hesperus* transcriptome and how this economically important insect pest responds to various abiotic stresses.

## Conclusions

Here, we report the elucidation of the adult *L. hesperus* transcriptome, which significantly enhances the molecular resources available for this arthropod pest. This transcriptional information not only provides a more nuanced understanding of the underlying biological and physiological mechanisms that govern *L. hesperus* biology, but may also lead to the identification of novel targets for biorationally designed control strategies. Among the transcripts identified were a number of putative Hsps, which are potentially crucial mediators of the *L. hesperus* cellular response to thermal stress. An increased understanding of how these molecular chaperones function in *L. hesperus* is essential for elucidating the phenotypic constraints on the ability of this insect to adapt to diverse environments. Furthermore, disruption of these gene products could potentially be exploited in a novel control strategy as RNA interference-mediated knockdown of Hsps in other insect species has been shown to have severe effects on dehydration tolerance [62], recovery from chill coma [63], and survival following thermal stress [64].

## Materials and Methods

### Insects

*Lygus hesperus* were from a laboratory colony reared at the USDA-ARS Arid Land Agricultural Research Center in Maricopa, AZ, USA. They were maintained on green beans and artificial diet [65], [66]. The colony was maintained at 25°C under 20% humidity and a L14: D10 photoperiod and annually outbred with local field-collected conspecifics to maintain vigor.

### RNA isolation and 454 sequencing

Total RNA was extracted from 20 mixed sex *L. hesperus* adults aged 0–5 days post-eclosion (5 each of 0–2 day old males and females and 3–5 day old males and females) using an RNeasy Plus Mini kit (Qiagen, Valencia, CA) according to the manufacturer's instructions. Total RNA quality was assessed on an Agilent BioAnalyzer 2100 with a RNA Nano 6000 LabChip Kit (Agilent Technologies, Santa Clara, CA) after DNase I (Qiagen) treatment. A 20 μg aliquot of the DNase-treated total RNA was shipped using RNAstable (Biomatrica, San Diego, CA) to the University of Illinois Urbana-Champaign Biotechnology Center (Urbana, IL) for normalized cDNA library construction. Messenger RNA was isolated from the total RNA using an Oligotex mRNA Mini kit (Qiagen). First and second strand cDNAs were synthesized from 200 ng mRNA using a SuperScript Double-Stranded cDNA Synthesis Kit (Invitrogen, Carlsbad, CA) with 100 μM random hexamer primers (Fermentas, Hanover, MD). Double-stranded cDNA was cleaned using a QIAquick MinElute PCR purification column (Qiagen) and nebulized using the kit supplied with the GS Titanium Library Preparation kit (454 Life Sciences, Branford, CT) according to the manufacturer's recommendations. Nebulized cDNA was blunt-ended, a 3′deoxyadenine-overhang was added, and adapters ligated. The resulting cDNA was size selected with AMPure beads (Agencourt Biosciences, Beverly, MA) and then amplified in 50 μl PCR reactions containing 10 μl 5x Phusion Buffer HF (New England Biolabs, Ipswich, MA), 25 μM Adapter A sense primer (5′CCATCTCATCCCTGCGTGTCTCCGACTCAGACGAGTGCGT3′), 25 μM Adapter B sense primer (5′CCTATCCCCTGTGTGCCTTGGCAGTCTCAGT3′), 10 μM dNTPs and 1 U Phusion polymerase (Finnzymes/New England Biolabs). The PCR conditions consisted of 98°C for 30 sec, followed by10 cycles of 98°C for 10 sec, 68°C for 30 sec and 72°C for 30 sec, with a final extension of 72°C for 5 min. The resulting products were cleaned using a QIAquick MinElute PCR column. The cDNA library was normalized according to the protocol described in the Trimmer Direct Kit (Evrogen, Russia). In brief, 300 ng of cDNA were incubated at 95°C for 5 min followed by incubation at 68°C for 4 hrs in the hybridization buffer included in the kit (50 mM Hepes, pH 7.5 and 0.5 M NaCl). After the incubation, the reaction was treated with a duplex specific nuclease and 1 μl was PCR amplified (12 cycles) as above. PCR products ranging from 400–1,000 bp were gel purified on a 2% agarose Ex-GEL (Invitrogen). Quantification of the normalized cDNA was performed on a Qubit fluorometer (Invitrogen) and the average size determined using an Agilent 2100 Bioanalyzer. An aliquot of the normalized library was sequenced on a Roche 454 GS FLX Titanium at the University of Hawaii Genomics Core Facility (Honolulu, HI) according to the manufacturer's recommendations.

### Bioinformatics

Prior to analyses, all adapter nucleotides were trimmed and the 454 sequences were *de novo* assembled using the newbler 2.6 software package (454 Life Sciences/Roche). The resulting isotigs were analyzed using BLASTx [67] against the GenBank non-redundant database with an *e*-value cut-off of 1*e* ^−5^. A tBLASTn analysis was performed using the most current (Sept, 2012) *L. hesperus* protein sequences. BLASTx-based comparative analyses of the *L. hesperus* transcriptome protein coding sequences were performed using putative protein sequences from *D. melanogaster*, *A. pisum*, and *P. humanus humanus*. Protein sequences shared across the species in question were determined using the online algorithm Venny (http://bioinfogp.cnb.csic.es/tools/venny/index.html). Homologous protein domains from translated *L. hesperus* transcriptomic sequences were identified by searching against the Pfam database [68] using HMMER3 [69]. Blast2GO [34], [35] was used to assign putative functionalities, GO terms, and KEGG (Kyoto Encyclopedia of Genes and Genomes) based metabolic pathways [38], [39]. Final GO assignments were defined based on a 10% filter for all three processes profiled at level 2. All other settings for the analysis were maintained at their defaults. For phylogenetic analysis, putative *L. hesperus* Hsp sequences and those of other insect species were aligned using MAFFT v6.814b [70], [71] as implemented in Geneious 5.6.5, and analyzed in MEGA 5 [72] using default maximum parsimony settings with 1000 bootstrap replications.

### Transcriptome mining and semi-quantitative PCR

To examine the affect of thermal stress on select Hsp genes, three biological replicates were maintained at 25°C (normal conditions), and three replicates were exposed to 39°C (thermal stress) for 6 hr. Each replicate consisted of a 6 day old adult female *L. hesperus* and all replicates were from a single cohort. Total RNA was obtained using TRI Reagent RNA Isolation Reagent (Ambion, Austin, TX) in conjunction with a TissueLyser (Qiagen) and 5 mm RNase Away-treated stainless steel beads. First-strand cDNA synthesis was performed using a RetroScript cDNA Synthesis Kit (Ambion) with 1 μg DNase I-treated total RNA and random decamer oligonucleotide primers as per the manufacturer's protocol. PCR amplimers (∼500 nt) of the selected Hsp transcripts were amplified using primers (Table 3) designed from the respective assembled isotig sequences. For control purposes, a fragment of the *L. hesperus* actin open reading frame (nt 1–554) was also amplified using primers (Table 3) designed to the *L. lineolaris* sequence (DQ386914). PCR was performed using ExTaq DNA polymerase premix (Takara-Clontech, Palo Alto, CA) with thermocycler conditions consisting of 95°C for 2 min followed by 27 cycles at 94°C for 20 sec, 56°C for 20 sec, and 72°C for 20 sec. Products were electrophoresed on a 1.5% agarose gel and visualized using SYBR Safe (Invitrogen). PCR amplification was performed on each of the biological replicates.

**Table 3:** Oligonucleotide primers used in semi-quantitative PCR and cloning.

Primer	Sequence (5′–3′)
LhHsp10 start F	ATG GCCAAAGCAACCGCAG
LhHsp10 end R	**TCA** CATTTCCAGTTTGGCGAGGATG
LhHsp21.5 start F	ATG GCAGACCAAGGTGTAAAG
LhHsp21.5 end R	**TTA** GTGCTGAGTGATAGGAATG
LhHsp21.4 start F	ATG TCTCTGTTGCCGATTG
LhHsp21.4 end R	**TCA** GGCTCCCTTCTTGTTC
LhHsp21.9 start F	ATG TCTCTGCTTCCGGCCC
LhHsp21.9 start F	**TTA** TTCAGCTGACGAGGCTG
LhHsp23.6 start F	ATG TCTCTGTTGCCAATTG
LhHsp23.6 end R	**TCA** GGCTCCCTTCTTGTTCTTC
LhHsp40-1 start F	ATG GGGAAGGATTATTACAA
LhHsp40-1 end R	**TTA** GGGCAACATGTCTCTCAG
LhHsp70-1 start F	ATG TCTGCTATTGGAATCG
LhHsp70-1 end R	**TTA** ATCAACTTCCTCGACAG
LhHsp90-1 start F	ATG CCGGAAGACGTAGAGATG
LhHsp90-1 end R	**TTA** GTCGACTTCTTCCATACG
LhHsp21.5 207 F	CTCCGTCATCGACACCGAGT
LhHsp21.5 660 R	CACGCCGTCTTTACTGAGCG
LhHsp21.4 11 F	TGCCGATTGTATTGAGCGAGC
LhHsp21.4 482 R	CCGGCTTTGAGCTCTTTGGG
LhHsp21.9 457 R	CAGCAATCGTAAGGACGCCG
LhHsp23.6 32 F	TCCTCAACGAGCGCCTCAAT
LhHsp23.6 494 R	CCGGCTTTGAGCTCTTTGGG
LhHsp40-1 236 F	GAGGTGGCCCTTCAGCTCAT
LhHsp40-1 721 R	TGTTCCTTCCCTGATCTCCTTCC
LhHsp70-1 984 F	GGGTCCATCCATGACGTGGT
LhHsp70-1 1455 R	GAATGCCGTTTGCGTCCAGA
LhHsp90-1 391 F	TCGCCCAGTTGATGTCCCTC
LhHsp90-1 864 R	TTCCTCGTCCAAGAGGCTCG
Lh actin 1 F	ATG TGCGACGAAGAAGTTG
Lh actin 555 R	GTCACGGCCAGCCAAATC

NOTE: putative start and stop codons are underlined and in bold font respectively.

To confirm the sequence of the assembled full-length isotigs, the respective Hsps were amplified from template cDNAs derived from the 6 hr 39°C samples using primers (Table 3) designed to amplify the respective ORFs and ExTaq DNA polymerase. PCR thermocycler conditions consisted of 95°C for 2 min followed by 35 cycles at 94°C for 30 sec, 56°C for 20 sec, and 72°C for 2 min, and a final 5 min extension at 72°C. Products were electrophoresed as before and amplimers of the expected sizes were gel excised, sub-cloned into the pGEM-T Easy cloning vector (Promega, Madison, WI), and DNA sequenced at the Arizona State University DNA Core Lab (Tempe, AZ).

### Data deposition

The *L. hesperus* Roche 454 sequence reads have been submitted to the NCBI Sequence Read Archive under the accession number SRA058144. Consensus nucleotide sequence data for the cloned *L. hesperus* Hsp DNA sequences have been deposited with GenBank under the following accession numbers JX627807-14.

## Supporting Information

Figure S1 **Comparative summary of the top 75 predicted Pfam protein domains from** ***L. hesperus*** **and predicted domains from** ***Aphis glycines*** [**20**] **and** ***Cimex lectularius*** [**32**] **.**
(EPS)

Figure S2 **Matrix describing the percent amino acid identity between the predicted** ***L. hesperus*** **Hsp10 and orthologous insect proteins.** The matrix is based on MAFFT alignment and includes partial sequences predicted in the *L. hesperus* transcriptome. Accession numbers are: EFN79770.1 (*Harpegnathos saltator*), EGI60182 (*Acromyrmex echinatior*), XP 624910.1 (*Apis mellifera*), XP_003691248.1 (*Apis florea*), XP_001599992.1 (*Nasonia vitripennis*), XP_002428683.1 (*Pediculus humanus humanus*), and XP_001848951 (*Culex quinquefasciatus*).
(EPS)

Figure S3 **Matrix describing the percent amino acid identity between the predicted** ***L. hesperus*** **sHsps and orthologous proteins from other insect species.** The matrix is based on MAFFT alignment and includes partial sequences predicted in the *L. hesperus* transcriptome. Accession numbers are: ABC84493 (*Locusta migratoria*), XP_973685 (*Tribolium castaneum*), ABC84494 (*Locusta migratoria*), AEV89760 (*Schistocerca gregaria*), XP 002423709 (*Pediculus humanus humanus*), ADK55524 (*Spodoptera litura*), EHJ69639 (*Danaus plexippus*), XP 973442 (*Tribolium castaneum*), and ACH85196 (*Bemisia tabaci*).
(EPS)

Figure S4 **Matrix describing the percent amino acid identity between the predicted** ***L. hesperus*** **Hsp40 proteins and orthologous proteins from other insect species.** The matrix is based on MAFFT alignment and includes partial sequences predicted in the *L. hesperus* transcriptome. Accession numbers are: ABF18277 (*Aedes aegypti*), ADD18658 (*Glossina morsitans morsitans*), EFZ09223 (*Solenopsis invicta*), XP_971446 (*Tribolium castaneum*), NP_001040292 (*Bombyx mori*), XP_002426657 (*Pediculus humanus humanus*), NP_001119620 (*Acyrthosiphon pisum*), and NP_001157382 (*Bombyx mori*).
(EPS)

Figure S5 **Matrix describing the percent amino acid identity between the predicted** ***L. hesperus*** **Hsp60 proteins and orthologous proteins from other insect species.** The matrix is based on MAFFT alignment and includes partial sequences predicted in the *L. hesperus* transcriptome. Accession numbers are: AAB94640 (*Culicoides variipennis*), NP_511115 (*Drosophila melanogaster*), EFN79769 (*Harpegnathos saltator*), XP_003700538 (*Megachile rotundata*), XP_002428684 (*Pediculus humanus humanus*), CAB58441 (*Myzus persicae*), XP_001951373 (*Acyrthosiphon pisum*), and AEV89752 (*Schistocerca gregaria*).
(EPS)

Figure S6 **Matrix describing the percent amino acid identity between the predicted** ***L. hesperus*** **Hsp70 proteins and orthologous proteins from other insect species.** The matrix is based on MAFFT alignment and includes partial sequences predicted in the *L. hesperus* transcriptome. Accession numbers are as follows: XP_002428084 (*Pediculus humanus humanus*), ACM78945 (*Spodoptera exigua*), NP_001153520 (*Apis mellifera*), ADE34170 (*Nilaparvata lugens*), AAP57537 (*Locusta migratoria*), AAZ17399 (*Bemisia tabaci*), XP_001945768 (*Acyrthosiphon pisum*), AFN02501 (*Bombyx mori*), EHJ78227 (*Danaus plexippus*), and BAM19890 (*Papilio xuthus*).
(EPS)

Figure S7 **Matrix describing the percent amino acid identity between the predicted** ***L. hesperus*** **Hsp90 proteins and orthologous proteins from other insect species.** The matrix is based on MAFFT alignment and includes partial sequences predicted in the *L. hesperus* transcriptome. Accession numbers are as follows: AAS45246 (*Locusta migratoria*), NP_001153536 (*Apis mellifera*), AFN02497 (*Tenebrio molitor*), XP_002428463 (*Pediculus humanus humanus*), ADK55517 (*Spodoptera litura*), AFG30049 (*Bombyx mori*), and XP_001861262 (*Culex quinquefasciatus*).
(EPS)

Table S1 **Results from a tBLASTn analysis of deposited** ***L. hesperus*** **proteins** (**Sept, 2012**) **against the adult** ***L. hesperus*** **transcriptome.**
(XLSX)

Table S2 **Top hits from a BLASTx search against the non-redundant protein database.** Analysis performed with an *e*-value cutoff of 1*e* ^−5^.
(XLSX)

Table S3 **Comparison of translated** ***L. hesperus*** **isotig sequences with those from Drosophila melanogaster,** ***Acyrthosiphon pisum*** **, and** ***Pediculus humanus humanus*** **.**
(XLSX)

Table S4 **Gene Ontology of** ***L. hesperus*** **transcriptomic sequences.**
(XLSX)

Table S5 **Summary of KEGG terms assigned to** ***L. hesperus*** **transcriptomic sequences.**
(XLSX)

Table S6 **Results of a Pfam domain search using** ***L. hesperus*** **transcriptomic sequences.**
(XLSX)
